# Case Report: Ictal Central Apnea as First and Overlooked Symptom in Temporal Lobe Seizures

**DOI:** 10.3389/fneur.2021.753860

**Published:** 2021-11-04

**Authors:** Elisa Micalizzi, Anna Elisabetta Vaudano, Giada Giovannini, Giulia Turchi, Leandra Giunta, Stefano Meletti

**Affiliations:** ^1^Department of Brain and Behavioral Sciences, University of Pavia, Pavia, Italy; ^2^Neurology Unit, Ospedale Civile di Baggiovara (OCB) Hospital, Azienda Ospedaliera-Universitaria, Modena, Italy; ^3^Department of Biomedical, Metabolic and Neural Science, University of Modena and Reggio Emilia, Modena, Italy; ^4^PhD Program in Clinical and Experimental Medicine, University of Modena and Reggio Emilia, Modena, Italy

**Keywords:** amygdala, temporal lobe epilepsy, seizures, SUDEP, ictal apnea

## Abstract

Ictal respiratory changes have been mainly described following generalized tonic-clonic seizures and recently considered to be a biomarker to assess the risk of sudden unexplained death in epilepsy (SUDEP). Nonetheless, modification of respiratory pattern can be related also to focal seizures, especially arising from the temporal lobe. Changes in cardiac function such as tachycardia or bradycardia could be often associated. We report a short case series of four patients with temporal lobe epilepsy admitted to our Epilepsy Monitoring Unit (EMU) presenting with an ictal central apnea as the first clinical manifestation of their seizures. None of these patients was aware of the occurrence of respiratory arrest. Age at onset ranged from 15 to 29 years. One patient had seizures with prolonged central apnea accompanied by a significant decrease in oxygen saturation. Neuroimaging in two patients showed alterations of mesial temporal lobe structures, including the amygdala. Recent neurophysiological studies supported the existence of a cortical network involving the limbic system that modulates downstream brainstem respiratory centers. Monitoring for respiratory changes and oxygen saturation in focal seizures is warranted for their potential value in identifying the epileptogenic zone and for a better understanding of ictal respiratory changes that could potentially define a subgroup of patients with high risk of seizure-related autonomic changes.

## Introduction

Peri-ictal autonomic changes might occur in epileptic seizures and are a potential biomarker for the risk of sudden unexplained death in epilepsy (SUDEP), which is the most common direct cause of death in patients with epilepsy (1). Both cardiac and respiratory dysfunction have been implicated as possible precipitating causes in SUDEP (2). Respiratory changes during epileptic seizures have been described in the literature (3, 4). Peri ictal respiratory changes are commonly related to bilateral tonic-clonic seizures (4) but can also be observed in focal seizures originating from the mesial structures of the temporal lobe. Desaturations and ictal central apnea (ICA) occurred most frequently in seizures arising from the temporal lobe (1, 2), and the longer the duration of the seizure, the higher the degree of desaturation. We report four cases of ICA as the first ictal clinical manifestation of seizures originating from the temporal lobe recorded in our epilepsy monitoring unit by means of a 10-20 EEG system integrated with EKG, SpO2, and thoracoabdominal bands for respiratory inductance plethysmography. According to published criteria, we considered as apnea a respiratory arrest of 5 or more seconds on the pneumographic channel; a desaturation was defined as a drop of SpO2 value below 95% (mild 90–94%, moderate 75–89%, severe <75%) (1, 5).

## Cases Description

### Case 1

**Figure 1:**
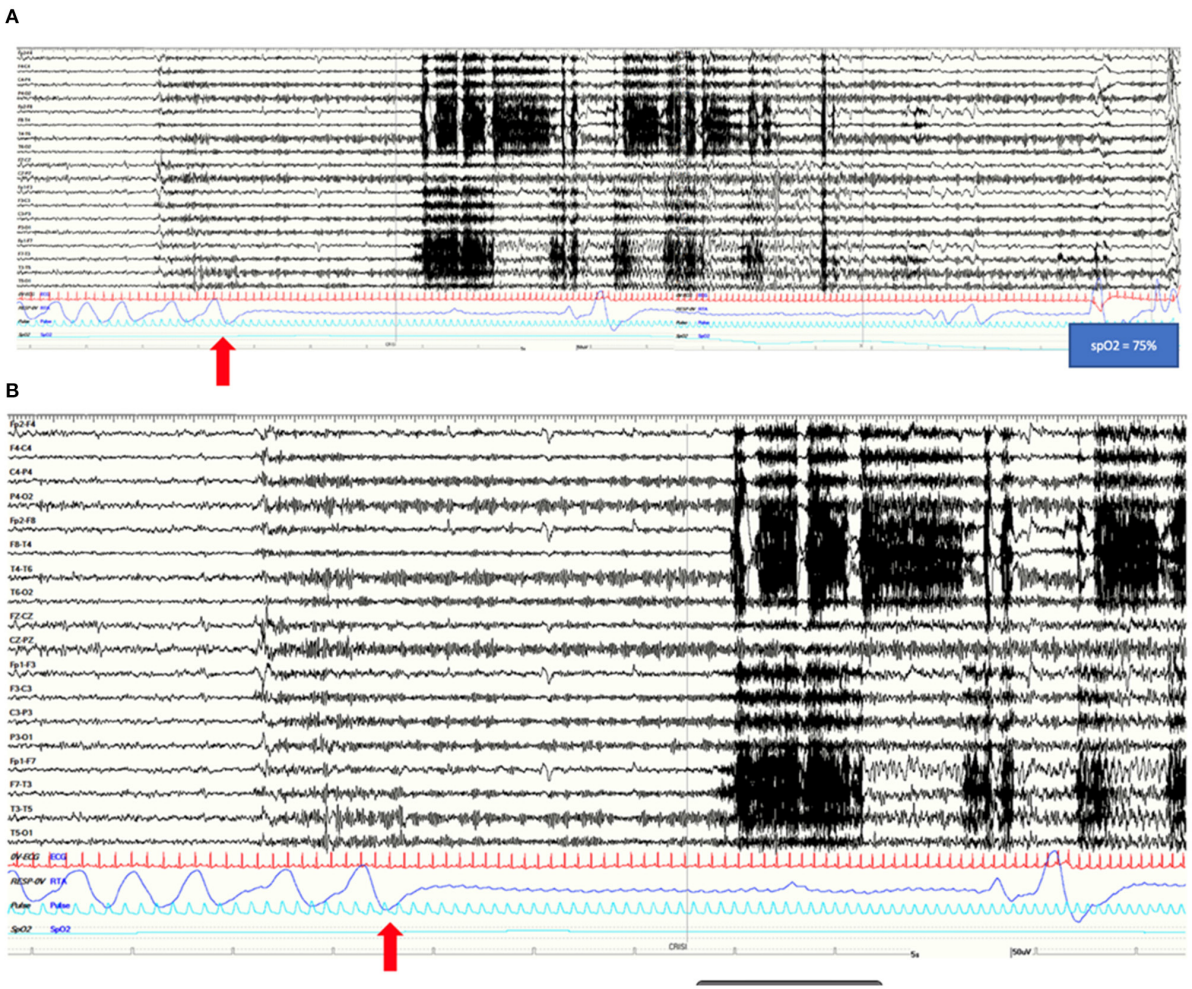
**(A)** A 2-min view of the EEG pattern with polygraphy of a left temporal lobe seizure characterized by rhythmic theta and delta activity on left fronto-temporal regions in patient 1. This long-lasting apnea induces a severe oxygen desaturation (SpO2 75%). **(B)** A more detailed view of the onset of apnea preceding EEG changes and EMG movement-related artifacts by 20 s. **(A,B)** Red arrows highlight the onset of the apnea. Note the ictal marked increase in heart rate. Red channel: ECG; blue channel: thoracoabdominal respirogram.

The patient is a right-handed 31-year-old female with no relevant past medical history and no family history of epilepsy. At the age of 25, she presented with two possible motor episodes during sleep and was started on antiseizure medication (ASM) with lamotrigine at a low dosage (50 mg/day). Around that time, she referred the occurrence of stereotyped subjective brief episodes characterized by forced thought, intense déjà-vu, anxious feeling associated with an ascending epigastric sensation. She would always be able to recall the events that tended to recur in brief clusters during the menstrual period. After 4 years of treatment, considering the persistence of these subjective episodes and the negative findings on interictal awake/sleep EEG recordings, as well as on 1.5 tesla brain MRI, lamotrigine was stopped in the hypothesis of non-epileptic seizures. A year later, she was admitted to our epilepsy monitoring unit where she underwent a long-term video-EEG monitoring (LTM) during wake and sleep. Routine laboratory findings as well as physical and neurological examinations were normal. A 3T brain MRI showed a slight hyperintensity of the anteromedial structures of the left temporal lobe. FDG-PET imaging was unremarkable. The interictal EEG showed sharp waves on left temporal region only during NREM and REM sleep. During LTM, we recorded three electroclinical seizures, two of them arising from sleep and one during wakefulness, characterized by ICA for around 17 s before any other clinical manifestation (Figure 1). In addition, the patient had impairment of awareness with disruption of contact, face pallor, and late oroalimentary automatisms. In each of the three recorded seizures, the respiratory disruption lasted for the entire ictal phase accompanied by drop of oxygen saturation (from 95% to a minimum of 75%) and mean delay between desaturation and clear EEG changes was of 22 s (Table 1). On the EEG, a left temporal ictal onset was observed. The patient was not aware of the apnea and did not present any respiratory distress. Lamotrigine was started again (50 mg/day) with complete control of seizures at a 5-month follow-up.

**Table 1:** Summary of apnea related findings in the described patients.

**Patient**	**Mean apnea duration (seconds)**	**Latency of apnea to first EEG changes (seconds)**	**Max desaturation**	**Awareness of apnea**	**Awareness impairment**	**Heart rate changes**	**Other ictal manifestations**
1	71.7	22	75%	No	Yes	Tachycardia	Psychomotor arrest, staring, oroalimentary automatisms
2	9.8	7.4	No desaturation	No	Yes	Tachycardia	Psychomotor arrest, staring, oromandibular automatisms, contralateral hand dystonia
3	16	1	89%	No	Not known	Tachycardia	Psychomotor arrest
4	19.5	9.5	92%	No	Yes	Tachycardia	Psychomotor arrest, late oromandibular automatisms

### Case 2

**Figure 2:**
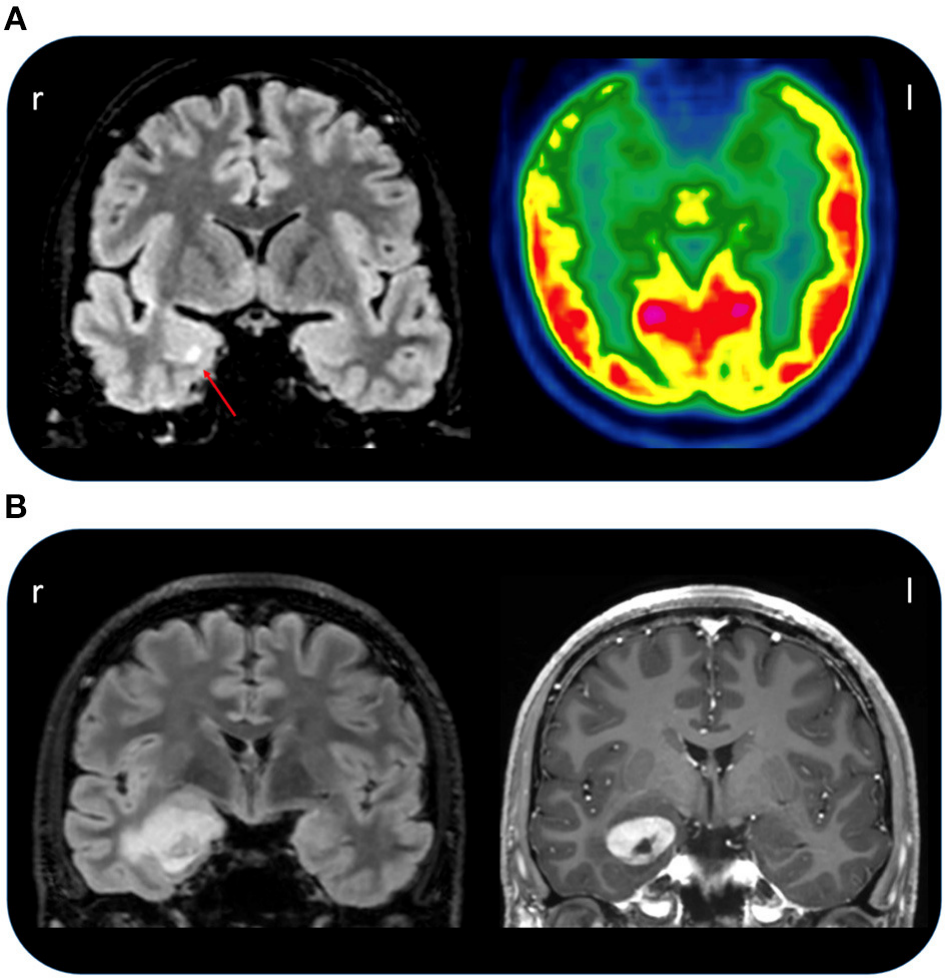
**(A)** Coronal FLAIR MRI of patient 2 (left) showing a hyperintense right amygdala with red arrow pointing to an area of increased signal intensity (red arrow). On the right, the FDG-PET image of the same patient showing right temporal hypometabolism involving the temporal pole and mesial regions. **(B)** Coronal FLAIR MRI of patient 4 (on the left) showing a temporo-mesial lesion that on T1 imaging (on the right) present a clear gadolinium enhancement suggesting a tumoral origin.

The patient is a left-handed 31-year-old male with a past medical history of puberal growth delay treated with GH hormone and negative family history for epilepsy. Since the age of 23, he manifested episodes characterized by sudden appearance of intrusive thoughts and epigastric discomfort followed by staring, unresponsiveness, and oroalimentary automatisms, occurring monthly in short clusters. Hence, in the hypothesis of epileptic seizures, lacosamide was started (up to 200 mg/day) with little or no benefit. In 2018, brain MRI showed a T2 FLAIR cortico-subcortical hyperintensity and T1 hypointensity of the right mesial temporal lobe (amygdala and uncus) without contrast enhancement (Figure 2A), interpreted as possible dysplasia or low-grade neuroglial lesion, that remained unvaried on subsequent follow-up. FDG-PET imaging showed right temporal lobe hypometabolism, especially of the temporal pole and of mesial structures (Figure 2A). The EEGs performed since the beginning of seizures were characterized by interictal slow abnormalities, spikes, and sharp waves over both left and right temporal derivations, occurring independently. In 2020, neuropsychological tests highlighted deficits of visuo-spatial memory and executive functions. He was admitted to our epilepsy monitoring unit in March 2021. Routine laboratory findings and physical and neurological examinations were unremarkable. He tested negative for anti-CNS antibodies on both serum and CSF. The interictal EEG confirmed spikes and sharp waves on left and right anterior temporal channels during wakefulness and sleep. A total of nine electroclinical seizures were recorded (2/9 during NREM sleep, 7/9 during wakefulness) arising from right anterior temporal channels. In 5/9 artifact-free seizures, ICA, lasting from 7 to 15 s, was recorded, as the first ictal clinical sign, followed by disruption of contact, staring, dystonic posturing of the left hand, oroalimentary automatisms, and eventually nose wiping with the right hand. He was unaware of the occurrence of these seizures and of the apnea. In this patient, no reduction of SpO2 was noticed. A time between 3 and 13 s from ICA onset elapsed before any observable EEG change, longer for the seizures occurring during NREM sleep. Tachycardia was present in all recorded seizures despite of the occurrence of respiratory arrest. He underwent right anterior temporal lobectomy with pathology showing aspecific gliosis. No seizures were reported at a 7-month follow-up.

### Case 3

A woman of 25 years, right-handed, reported the occurrence of two episodes of loss of consciousness with motor features during sleep at the age of 15. A first brain MRI was normal. Relying on clinical features, epilepsy was suspected and levetiracetam was started (up to 1,500 mg/day) with consistent efficacy. At the age of 16, during wakefulness, she manifested rare episodes characterized by impairment of awareness, staring, language disturbances, and hypersalivation followed by spontaneous recovery after 1 or 2 min. Right after, she was able to speak properly but not to recall the event. Then, lamotrigine was started (up to 300 mg/day) in association with levetiracetam with slight reduction in frequency of these episodes. After 4 years of seizure freedom, brief but frequent stereotyped episodes with the same features as described above recurred. Lacosamide was added in polytherapy (200 mg/day) without seizures' remission. A second brain MRI (3T) and the FDG-PET were unremarkable. The EEGs performed during the years showed frequent interictal spikes and slow waves over the left temporal lobe derivations. General and neurological examinations were normal. She was admitted to our monitoring unit where we recorded one seizure arising from the left temporal lobe characterized by ICA lasting 23 s, preceding by one second the EEG ictal onset with subsequent moderate desaturation to SpO2 89% and psychomotor arrest (Table 1). The patient was unaware for the occurrence of ictal modification of breathing and showed no respiratory distress.

### Case 4

**Figure 3:**
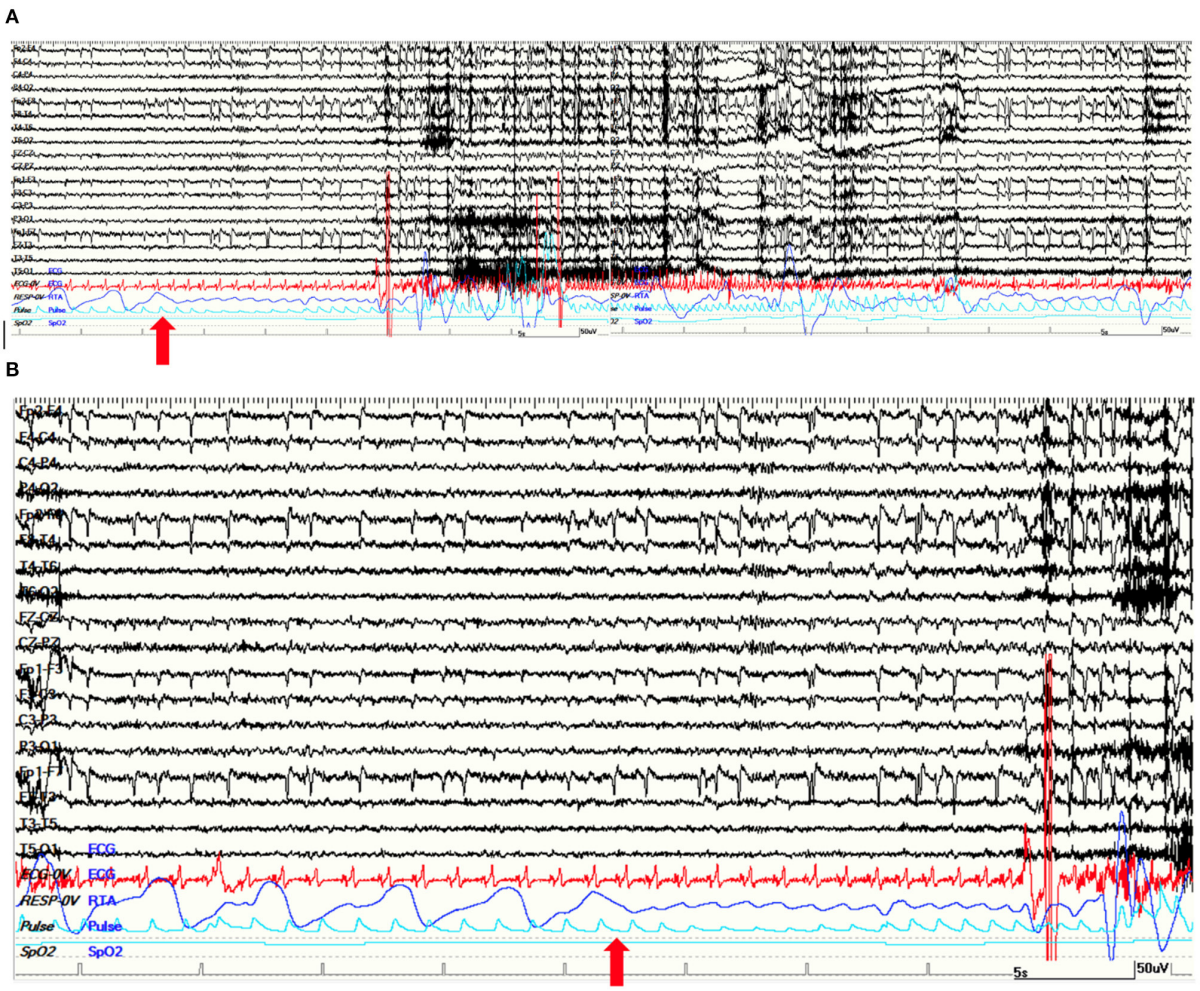
**(A)** A 120-s view of a right temporal lobe seizure of patient 4. The apnea was the first ictal sign. The ictal EEG is characterized by a slow rhythmic theta activity on right temporal regions. **(B)** Apnea started 15 s before first EEG changes. **(A,B)** Red arrows point at the beginning of apnea on thoraco-abdominal polygraphic channel. Note the ictal marked increase in heart rate. Red channel: ECG; blue channel: respirogram.

This patient is a 31-year-old male, left-handed, with no relevant past medical history except for the recurrence of stereotyped episodes characterized by brief déjà-vu sensations along with a mild headache in the last 2 years. He was admitted to our monitoring unit after a bilateral tonic-clonic seizure. Neurological and general examinations were unremarkable. Laboratory findings were normal. The 1.5 T MRI showed a right mesial temporal cortico-subcortical lesion (amygdala, temporal uncus, hippocampal head, and part of the hippocampal body) with gadolinium enhancement (Figure 2B). He underwent video-EEG LTM that showed rare spikes and sharp waves over the fronto-temporal regions during NREM sleep. We recorded four seizures arising from the right temporal regions during wakefulness, with occurrence of central apnea (lasting from 15 to 40 s) preceding the first EEG changes by 6–27 s (Figure 3). A mild ictal desaturation (SpO2 nadir 92%) was noted in all seizures. Clinically, the patient did not warn and manifested psychomotor and respiratory arrest, late oroalimentary, and some motor automatisms; when asked, he reported a mental picture which he could not recall but that seemed to be familiar to him. Ictal increase of heart rate was always observed. He was unaware of the occurrence of the apnea and did not report dyspnea. Lacosamide was started (until 200 mg/day); subsequently, the patient was addressed to surgery to perform an anterior temporal lobectomy. Pathology revealed a ganglioglioma, a grade I neuronal-glial tumor according to the 2016 CNS WHO classification. The patient appeared seizure-free at a 12-month follow-up.

## Discussion

Here, we reported four patients with TLE whose seizures were characterized by an ictal central apnea of variable duration that preceded, or was concomitant, with the scalp-EEG recorded seizures (see Table 1). The first description of a respiratory disruption in a patient experiencing focal seizure belongs to Jackson (6). A systematic review and meta-analysis of 21 studies (4) found an incidence of ictal hypoxemia around one third of seizures, with higher estimates found for adult patients presenting with tonic-clonic seizures. Severe peri-ictal desaturation (below 75% of SpO2) were associated to higher risk of SUDEP, considering that respiratory dysfunctions are correlated with increased risk of fatal cardiac rhythm abnormalities (7, 8). While data on peri-postictal apnea in tonic-clonic seizures are well-known, little attention has been paid until recent years toward ictal apnea in focal non-motor/non convulsive seizures. Here, we described four very similar cases in whom ICA was the first ictal symptoms in seizures involving temporo-limbic networks.

Data suggesting a functional connection between the amygdala and neural networks in the brainstem that control the involuntary respiratory drive derive from studies conducted on animals, such as cats (9), rabbits (10), and primates (11). As regards this functional connection between amygdala and brainstem in humans, the only data available come from clinical evidence, especially from invasive neurophysiological and neurosurgical studies. In 1952, Kaada and Jasper reported that intraoperative stimulation of different targets in the temporolimbic cortex-provoked apnea in their subjects (12). In 1954, Penfield and Jasper demonstrated that intraoperative stimulation of the cingulate cortex, rolandic cortex, and uncal regions could also generate brief apneic responses (13). Afterwards, in 1972, Bonvallet and Bobo noted that intraoperative stimulation of the amygdala was associated with apnea and bradycardia (9). Dlouhy et al. (14) documented with intracranial electrodes in one patient that apnea conditioning desaturation could be elicited by seizure spread to basolateral and lateral amygdala (left side) and in three patients upon direct stimulation of these nuclei (both left and right). As it occurred in our patients, none of them was aware of ictal respiratory modification nor appeared distressed. They observed a reduction in heart rate, differently from our four patients who presented tachycardia. They also assessed that unilateral amygdala stimulation is sufficient to cause apnea. More recently, Lacuey et al. (15) reported an incidence of ICA of 68.7% in their patients with mesial temporal lobe seizures, suggesting that this can be a common feature of temporal lobe epilepsy (TLE). They also noted that ICA is often the first clinical sign and sometimes the only clinical manifestation in medial TLE, suggesting its relevance in the anatomo-electro-clinical localization of the seizure onset zone. In another study, Lacuey et al. (15) investigated nineteen consecutive adult patients undergoing stereotactic EEG recordings and elicited a central apnea in 13/19 patients by electrical stimulation of the amygdala, hippocampus head and body, anterior parahippocampal gyrus, and antero-mesial fusiform gyrus. Therefore, they concluded that might exist a limbic and paralimbic network involved in the respiratory regulation of brainstem respiratory centers. Notably, amygdala stimulation might induce functional deafferentation of brainstem structures impairing involuntary breathing controlled by brainstem connections.

The apnea agnosia observed in our patients could be the reason why ICA in focal seizures is often unrecognized by clinicians. Different physiopathological mechanisms underlying this phenomenon have been proposed (14): (i) amygdala stimulation is thought to inhibit forebrain structures (insula or cingulate cortex) that are implicated in the conscious perception of dyspnea (16); (ii) amygdala stimulation can inhibit CO2/pH-sensitive serotoninergic neurons in the midbrain that mediate CO2-evoked arousal (17, 18); (iii) the absence of apnea awareness could be related to the effect of ictal activity on midbrain structures implicated in maintaining arousal/vigilance levels.

Regardless of the pathophysiologic mechanisms underlying ICA, the lack of awareness of apnea could lead to increased risk of SUDEP, especially while sleeping in prone position when self-defense response to respiratory distress could be fundamental (14). ICA is usually a self-limited event but, in some cases, can be prolonged and might be responsible of unobserved death in patients with epilepsy (1). Of course, at present, no data can link ICA to an increased risk of SUDEP; however, ICA could define a subgroup of patients with high-risk of seizure-related autonomic changes that warrant to be studied in more detail. Another interesting finding in our small case series is that apnea precede the scalp-EEG ictal changes by many seconds (up to 20 s in patient 1), suggesting that seizure activity can remain very localized for such a time before spreading to other brain regions, as reported in studies that combined scalp and intracranial EEG (15, 19). Hence, in the absence of an adequate polygraphic monitoring, seizures might go unrecognized, especially if the apnea is the only clinical manifestation. For this reason, we advocate recordings of respiratory changes and oxygen saturation in long-term monitoring of epilepsy patients, that in our view should become a “standard of care” in the context of the epilepsy monitoring unit.

Limitations of our findings may be due to the limited number of our patients and the lack of recordings of intracranial EEG activity that could give more precise information about the symptomatogenic zone of the seizure network. However, we would suggest monitoring of respiratory parameters, such as SpO2 and respiratory inductance plethysmography, also in a diagnostic and pre-surgical work up in patients with focal epilepsy. Data obtained from this type of monitoring could be useful to better localize the epileptogenic zone and to assess potential seizure-related biomarkers of increased SUDEP risk.

## Data Availability Statement

The original contributions presented in the study are included in the article/supplementary material, further inquiries can be directed to the corresponding author/s.

## Ethics Statement

The studies involving human participants were reviewed and approved by Comitato Etico Area Vasta Emilia Nord. The patients/participants provided their written informed consent to participate in this study.

## Author Contributions

All authors listed have made a substantial, direct and intellectual contribution to the work, and approved it for publication.

## Funding

This work was supported by Dipartimento di eccellenza 2018-2022, MIUR, Italy, to the Department of Biomedical, Metabolic and Neural Sciences, University of Modena and Reggio Emilia; Ricerca Finalizzata, project code NET-2013-02355313, Ministry of Health to the Azienda Ospedaliera-Universitaria di Modena Centro hub chirurgia epilessia (DGR 1172/18).

## Conflict of Interest

The authors declare that the research was conducted in the absence of any commercial or financial relationships that could be construed as a potential conflict of interest.

## Publisher's Note

All claims expressed in this article are solely those of the authors and do not necessarily represent those of their affiliated organizations, or those of the publisher, the editors and the reviewers. Any product that may be evaluated in this article, or claim that may be made by its manufacturer, is not guaranteed or endorsed by the publisher.

## References

[1] Vilella L Lacuey N Hampson JP Rani MRS Loparo K Sainju RK. Incidence, recurrence, and risk factors for peri-ictal central apnea and sudden unexpected death in epilepsy. Front Neurol. (2019) 10:166. 10.3389/fneur.2019.00166 30890997 PMC6413671

[2] Bateman LM Li CS Seyal M. Ictal hypoxemia in localization-related epilepsy: analysis of incidence, severity and risk factors. Brain. (2008) 131:3239–45. 10.1093/brain/awn277 18952672 PMC2639205

[3] Blum AS. Respiratory physiology of seizures. J Clin Neurophysiol. (2009) 26:309–15. 10.1097/WNP.0b013e3181b7f14d 20168130

[4] Bruno E Maira G Biondi A Richardson MP on behalf of the RADAR-CNS Consortium. Ictal hypoxemia: a systematic review and meta-analysis. Seizure Eur J Epilepsy. (2018) 63:7–13. 10.1016/j.seizure.2018.10.011 30391664

[5] Lacuey N Zonjy B Hampson JP Sandhya Rani MR Zaremba A Sainju RK. The incidence and significance of peri-ictal apnea in epileptic seizures. Epilepsia. (2018) 59:573–82. 10.1111/epi.14006 29336036 PMC6103445

[6] Jackson JH. On Asphyxia in slight epileptic paroxysms: On the symptomatology of slight epileptic fits supposed to depend on discharge-lesions of the uncinate gyrus. Lancet. (1899) 1:79–8.

[7] Kiely DG Cargill RI Grove A Struthers AD Lipworth BJ. Abnormal myocardial repolarization in response to hypoxaemia and fenoterol. Thorax. (1995) 50:1062–6. 10.1136/thx.50.10.1062 7491554 PMC475019

[8] Roche F Reynaud C Pichot V Duverney D. Effect of acute hypoxia on QT rate dependence and corrected QT interval in healthy subjects. Am J Cardiol. (2003) 91:916–9. 10.1016/S0002-9149(03)00040-7 12667592

[9] Bonvallet M Bobo EG. Changes in phrenic activity and heart rate elicited by localized stimulation of amygdala and adjacent structures. Electroencephalog Clin Neurophysiol. (1972) 32:1–16. 10.1016/0013-4694(72)90223-4 4109912

[10] Applegate CD Kapp BS Underwood MD McNall CL. Autonomic and somatosensory effects of amygdala central N. stimulaton in awake rabbits. Physiol Nehav. (1983) 31:353–60. 10.1016/0031-9384(83)90201-9 6635005

[11] Reis DJ McHugh PR. Hypoxia as a cause of bradycardia during amygdala stimulation in monkey. Am J Physiol. (1968) 214:601–10. 10.1152/ajplegacy.1968.214.3.601 4966348

[12] Kaada BR Jasper H. Respiratory responses to stimulation of temporal pole, insula, and hippocampal and limbic gyri in man. AMA Arch Neurol Psychiatry. (1952) 68:609–19. 10.1001/archneurpsyc.1952.02320230035004 12984874

[13] Penfield W Jasper H. Epilepsy and the Functional Anatomy of the Human Brain. Boston: Little, Brown and Company (1954). p. 830–1.

[14] Dlouhy BJ Gehlbach BK Kreple CJ Kawasaki H Oya H Buzza C. Breathing inhibited when seizures spread to the amygdala and upon amygdala stimulation. J Neurosci. (2015) 35:10281–9. 10.1523/JNEUROSCI.0888-15.2015 26180203 PMC4502266

[15] Lacuey N Hampson JP Harper RM Miller JP Lhatoo D. Limbic and paralimbic structures driving ictal central apnea. Neurology. (2019) 92:e655–69. 10.1212/WNL.0000000000006920 30635481 PMC6382368

[16] Buchanan GF Richerson GB. Role of chemoreceptors in mediating dyspnea. Respir Physiol Neurobiol. (2009) 167:9–19. 10.1016/j.resp.2008.12.002 19118647 PMC4486073

[17] Buchanan GF Richerson GB. Central serotonin neurons are required for arousal to CO2. Proc Natl Acad Sci USA. (2010) 107:16354–9. 10.1073/pnas.1004587107 20805497 PMC2941296

[18] Severson CA Wang W Pieribone VA Dohle CI Richerson GB. Midbrain serotonergic neurons are central pH chemoreceptors. Nat Neurosci. (2003) 6:1139–1140. 10.1038/nn1130 14517544

[19] Lacuey N Hupp NJ Hampson J Lhatoo SD. Ictal Central Apnea (ICA) may be a useful semiological sign in invasive epilepsy surgery evaluations. Epilepsy Res. (2019) 156:106164. 10.1016/j.eplepsyres.2019.106164 31330483

